# Reproductive effort and future parental competitive ability: A nest box removal experiment

**DOI:** 10.1002/ece3.4342

**Published:** 2018-08-11

**Authors:** Rienk W. Fokkema, Richard Ubels, Christiaan Both, Livia de Felici, Joost M. Tinbergen

**Affiliations:** ^1^ Conservation Ecology Group Groningen Institute for Evolutionary Life Sciences (GELIFES) University of Groningen Groningen The Netherlands; ^2^ Evolutionary Biology Bielefeld University Bielefeld Germany; ^3^ Department of Animal Behaviour Bielefeld University Bielefeld Germany

**Keywords:** brood size manipulation, carry‐over effects, intraspecific competition, life history theory, social environment

## Abstract

The life history trade‐off between current and future reproduction is a theoretically well‐established concept. However, empirical evidence for the occurrence of a fitness cost of reproduction is mixed. Evidence indicates that parents only pay a cost of reproduction when local competition is high. In line with this, recent experimental work on a small passerine bird, the Great tit (*Parus major*) showed that reproductive effort negatively affected the competitive ability of parents, estimated through competition for high quality breeding sites in spring. In the current study, we further investigate the negative causal relationship between reproductive effort and future parental competitive ability, with the aim to quantify the consequences for parental fitness, when breeding sites are scarce. To this end, we (a) manipulated the family size of Great tit parents and (b) induced severe competition for nest boxes among the parents just before the following breeding season by means of a large‐scale nest box removal experiment. Parents increased their feeding effort in response to our family size manipulation and we successfully induced competition among the parents the following spring. Against our expectation, we found no effect of last season's family size on the ability of parents to secure a scarce nest box for breeding. In previous years, if detected, the survival cost of reproduction was always paid after midwinter. In this year, parents did pay a survival cost of reproduction before midwinter and thus before the onset of the experiment in early spring. Winter food availability during our study year was exceptionally low, and thus, competition in early winter may have been extraordinarily high. We hypothesize that differences in parental competitive ability due to their previous reproductive effort might have played a role, but before the onset of our experiment and resulted in the payment of the survival cost of reproduction.

## 1 INTRODUCTION

One of the corner stones of life‐history theory is the cost of reproduction: an increase in current reproduction goes at the expense of fitness that will be gained from future reproduction (Barnes & Partridge, 2003; Williams, 1966). Parents with high reproductive investment are expected to pay a cost either via a decreased survival probability, a reduced future fecundity, or both. The higher the fitness costs of reproduction, the more the parents are selected to lower their reproductive investment (Roff, 1992; Stearns, 1992; Tinbergen and Daan, 1990). Empirically, the evidence for the occurrence of fitness costs of reproduction is ambiguous. Although negative effects of reproductive investment on future reproduction like second or repeated reproductive attempts of the parent in the same season are relatively well established, the evidence for specifically a survival cost of reproduction is mixed (birds: Dijkstra et al., 1990; Golet, Irons, & Estes, 1998; Linden & Moller, 1989; Parejo & Danchin, 2006; Santos & Nakagawa, 2012; Stearns, 1992; mammals: Hamel et al., 2010; Stearns, 1992).

Fitness costs of reproduction may be mediated both by physiology or ecology or a combination of both (Lessels, 1991; Speakman, 2008; Zera & Harshman, 2001). The focus of most studies has primarily been on physiological mechanisms behind fitness costs of reproduction. These studies show that reproductive effort entails physiological costs as depletion of energy stores, depletion of micronutrients, physiological stress, oxidative stress, immunosuppression, and costs to maintain neuroendocrine control systems (discussed in: Alonso‐Alvarez & Velando, 2012). While these physiological mechanisms tell us something about how the cost of reproduction are being paid, they do not answer why in some study populations costs of reproduction have been detected and not in others. Knowledge of the ecological mechanisms likely provides more insight in the occurrence of costs of reproduction.

Studies on ecological selection pressures behind the occurrence of fitness costs of reproduction have focused mostly on the relationship between reproductive effort and the predation risk of parents and their offspring (Alonso‐Alvarez & Velando, 2012; Fontaine & Martin, 2006; Lessels, 1991; Magnhagen, 1991; Martin, Scott, & Menge, 2000; Roff, 1992). Intra‐ and interspecific competition is a major ecological selection pressure that, along with predation, could mediate the costs of reproduction. In many populations, a negative relationship between clutch or litter size and population density has been found (Both, Tinbergen, & Visser, 2000; Dhondt, Kempenaers, & Adriaensen, 1992; Kluijver, 1951; Newton, 1998; Nicolaus, Brommer, Ubels, Tinbergen, & Dingemanse, 2013; Perrins, 1965; Sedinger & Lindberg, 1998; but see: Alatalo & Lundberg, 1984; mammals: Bonenfant et al., 2009; Koskela, Mappes, & Ylönen, 1999; Morris, 1989). Several proximate mechanisms have been formulated to explain these density dependent effects on family size, most of them related to higher levels of competition for resources such as food or territories at high population densities (Kluijver, 1951; Newton, 1998; Tinbergen, Van Balen, & Van Eck, 1985). Correlational evidence shows that the costs of reproduction are higher at high population density and presumably competition (Festa‐bianchet, Gaillard, & Jorgenson, 1998; Oksanen, Koivula, Koskela, & Mappes, 2007). Due to increased costs of reproduction at high population density parents may be under selection to produce smaller families. Experimental studies in which the occurrence of fitness costs of reproduction under competition is investigated are, however, scarce.

Testing whether costs of reproduction occur and whether they are indeed modulated by the competitive environment involves experimentally manipulating not only the reproductive investment of a parent, but also the level of competition in the parents’ environment. This was performed by Nicolaus et al. (2012) who simultaneously manipulated the family size that the small passerine the Great tit (*Parus major*) had to raise and the local levels of intraspecific competition. Subsequently, they measured the existence of fitness cost of reproduction. Nicolaus et al. (2012) found that survival costs of reproduction were only paid in environments with high levels of competition. These survival effects occurred after midwinter and thus well after the breeding season. The level of competition within a parents’ (future) social environment may thus be an important determinant of whether or not it pays a survival cost of reproduction. Nicolaus et al. (2012) hypothesized that family size may negatively affect the competitive ability of parents. Earlier work by Siefferman and Hill (2005a) indicated that this may indeed be the case. Male Eastern bluebirds (*Sialia Sialis*) that raised experimentally reduced families had significantly brighter plumage in the following year and were able to mate with females, of better condition and/or with more experience, who initiated egg laying earlier in the season. Males with significantly brighter plumage were as the authors hypothesized based on previous work better able to compete for nest cavities and therefore more attractive to these “higher quality” females (Siefferman & Hill, 2005b). Through such processes, males with reduced reproductive effort may have achieved higher future reproductive success.

Within a nest box breeding Great tit population, we put the potential negative effect of family size on the future competitive ability of parents as hypothesized by Nicolaus et al. (2012) further to the test. We measured the long‐term effect of manipulated family size on the ability of Great tit parents to compete in the ensuing winter for roosting boxes (Fokkema, Ubels, & Tinbergen, 2017) and in spring for preferred deeper nest boxes (Fokkema, Ubels, & Tinbergen, 2016). In the winter period, we found no evidence for a negative effect of family size on the ability of parents to claim a roosting box, but in spring we did find that manipulated family size negatively affected the ability of Great tit parents to claim a preferred deeper nest box. In a follow‐up study focused on Blue tits (*Cyanistes caeruleus*), we found that deeper nest boxes offered higher breeding success, especially in areas with high predation pressure (Fokkema et al. in press). Hence, our experiments supported the hypothesis that a higher reproductive effort reduced parental competitive ability in the next breeding season, which could potentially reduce parental fitness under high competition.

One difficulty we ran into in our previous work is that we could not exclude the possibility that parents may have been differentially motivated to compete for resources depending on their previous reproductive effort (see Discussion: Fokkema et al., 2016). Parents may as a consequence of their family size manipulation have differed in their future reproductive potential and this could have affected their motivation to compete. Such motivational differences could be explained by the terminal investment hypothesis (Billing, Rosenqvist, & Berglund, 1999; Bonneaud, Mazuc, Chastel, Westerdahl, & Sorci, 2004; Creighton, Heflin, & Belk, 2009; Velando, Drummond, & Torres, 2006; Williams, 1966) and/or the asset protection principle (Clark, 2000; Wolf, van Doorn, Leimar, & Weissing, 2007). For example, in our experiment in which we induced competition for deeper nest boxes, our findings could be explained by the asset protection principle. Parents who raised reduced families may have been more inclined to compete for the deeper nest boxes which were safer from predation in order to safeguard their future reproductive potential (their future “asset”; Fokkema et al., 2016).

In our current study, to overcome such motivational differences among parents, we aimed to induce competition for a resource that we expected all parents would be highly motivated to compete for. During the breeding season, we manipulated the family size that Great tit parents had to raise and just before the onset of the following breeding season, we drastically reduced the number of nest boxes available for breeding in the whole study area. We expected that all Great tit parents irrespective of their previous reproductive effort would be motivated to compete for a breeding box as not breeding for the short‐lived Great tit is not really an option (See life table in: Tinbergen and Daan, 1990). We expected that there were only limited and nonpreferred alternative natural breeding cavities available for the Great tits. Nest boxes are generally preferred by Great tits over natural cavities (Drent, 1987; Lõhmus & Remm, 2005). Further, in our study area, the availability of natural cavities was low as the woods were relatively young (planted in 1969, Newton, 1994). Competition in our study was thus more a zero‐sum game, as failing to obtain a nest box meant that individuals unlikely could breed. Therefore, we expected that the fitness consequences of reduced competitive ability would be much more severe if the parents had to compete to breed relative to our previous work in which we manipulated the quality, but not the quantity of nest boxes (Fokkema et al., 2016). This would make the impact of family size on fitness larger, and in addition the so generated cost of reproduction would more clearly interact with population size relatively to the available resources (nest boxes). In that way, selection for smaller broods at higher densities would be expected.

## 2 MATERIALS AND METHODS

### 2.1 Study area

**Figure 1:**
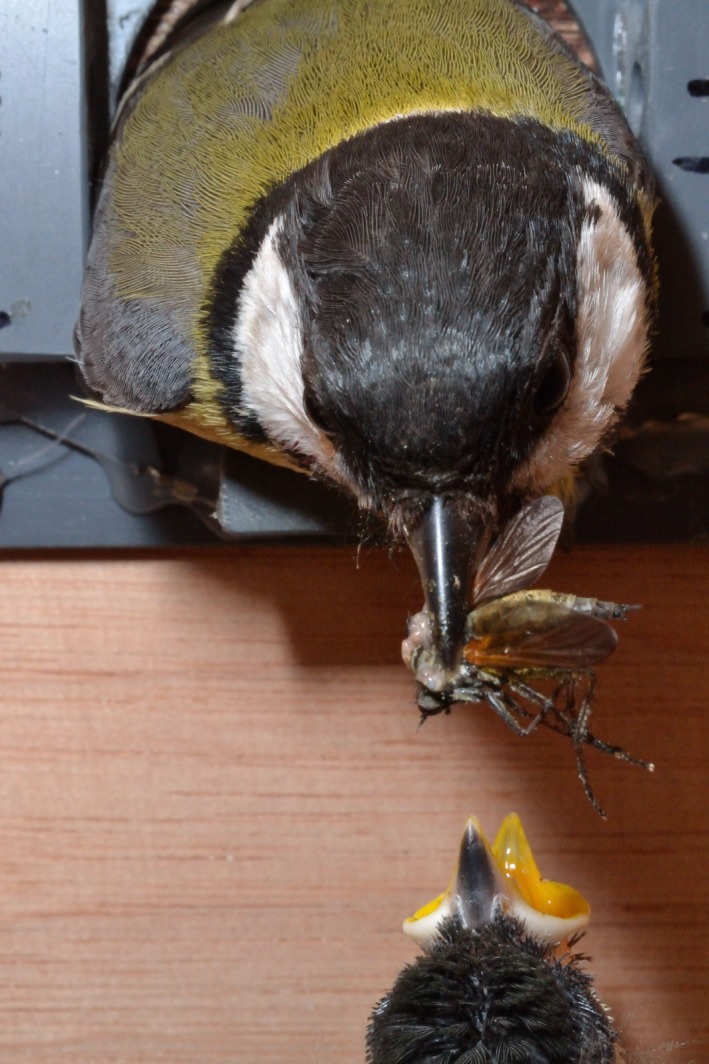
A Great tit (Parus major) feeding its nestlings. In this study, we manipulated the feeding effort of Great tit parents by manipulating the size of the family parents had to raise. Picture by: Rienk Fokkema [Colour figure can be viewed at http://wileyonlinelibrary.com]

The study was carried out in a nest box breeding population of Great tits in the Lauwersmeer area, in the northern part of the Netherlands (53°23′N, 6°14′E; for study species see: Figure 1). The study area was reclaimed from the Wadden sea in 1969 after which parts were planted with deciduous trees and some conifers. The study area consisted of 12 plots of roughly 10 ha distributed over the forests (for map see: Nicolaus et al. 2009). Before the nest box removal experiment, each plot contained 50 nest boxes attached to trees at breast height (approx. 1.20 m), separated 50 m from each other in a grid. The 600 wooden nest boxes were made of 2 cm thick plywood with inside dimensions of approximately: length, width, height: 12, 8, 24 cm.

### 2.2 Manipulation of parental feeding effort

#### 2.2.1 Family size manipulation

In 2014, we closely monitored all nest boxes during the breeding season to determine the start of egg laying. As soon as incubation had commenced (warm, uncovered eggs in the nest cup), we calculated the expected hatching date, based on the first egg laying date and the clutch size. We assumed that one egg was laid per day and incubation took at least 12 days (Fokkema et al., 2016). Starting 2 days before the expected hatching date, nests were checked daily until the first egg hatched. Based on the data gathered when the nestlings were 5 days old, we formed “trios” of nests with similar initial conditions. Nests within a trio had in order of importance: (a) the exact same hatching date, (b) a similar brood size when the nestlings were 5 days old (0–1 nestlings difference), (c) a similar clutch size (0–2 eggs difference), and (d) an approximately similar brood weight when the nestlings were 5 days old (1–24 g difference). Overall this resulted in a highly similar background for the manipulation groups (see: Table 1). Within these trios, we performed our family size manipulations when the nestlings were 6 days old. Nest treatment and nestlings to be exchanged were randomly assigned. Within most matched trios, we exchanged three nestlings from one brood (reduced) to another brood (enlarged) and kept a third nest as control (34 trios). To disturb each nest to a similar extent, we also exchanged two nestlings of the reduced brood to the control brood and vice versa and repeated this procedure for the enlarged brood. For three matched trios, we exchanged two nestlings instead of three to prevent desertion of the brood by the parents (initial family size of trios: 6–7 nestlings; Verboven et al. 2002; for further details on manipulation scheme, see: Fokkema et al., 2016; de Jong et al. 2014). The average age of the parents that raised the manipulation groups was similar (Reduced: 1.32, Control: 1.38, Enlarged: 1.31, on a scale of 1 being a first year breeding bird and 2 an experienced breeder).

**Table 1:** Family size manipulations were performed within a trio of matched nests, in which either 2 (−2/0/+2) or 3 nestlings were exchanged (−3/0/+3). Column 2 and 3 show the sample size and the mean original clutch size. Column 4 and 5 show the mean number of nestlings‐ and brood weight before the family size manipulation at day 6. CI, confidence interval

No. of nestlings exchanged	*N*	Clutch size (CI)	No. of nestlings (CI)	Brood weight (g) (CI)
−2	3	7.00 (2.48)	6.33 (1.43)	42.33 (27.36)
0	3	6.67 (2.87)	6.00 (0.00)	41.80 (9.02)
2	3	6.67 (1.43)	6.67 (1.43)	46.87 (8.10)
−3	34	9.29 (0.34)	8.97 (0.38)	60.08 (3.50)
0	34	9.24 (0.34)	8.88 (0.29)	59.60 (2.76)
3	34	9.26 (0.30)	8.79 (0.36)	59.82 (3.85)

When the nestlings were 7 days old, we attempted to catch all parents that raised a manipulated brood (reduced, control, or enlarged; here termed “manipulated parents”) using spring traps and provide them with a unique ring combination: a differentially colored RFID transponder (type: EM4102 bird PIT tag 2.6 mm, manufactured by: IB technology, Eccel Technology Limited) on one leg in combination with a plastic color ring and metal ring with inscription on the other leg. This allowed us to identify the birds (a) when measuring the number of feeding visits, (b) at potential late broods within the same season, (c) during our winter and early‐spring roost checks, and (d) in our early spring observation sessions (see below).

#### 2.2.2 Components of feeding effort

We measured the effect of family size manipulation on four components related to parental feeding effort: (a) the number of feeding visits per day by each parent to the nest box when the nestlings where 12 days old (the nestling age around which brood energy demand peaks: van Balen 1973; Sanz and Tinbergen 1999; Tinbergen and Dietz 1994; using a reader equipped to detect the RFID transponders of the manipulated parents: LID665, version V804, manufactured by Dorset identification b.v), (b) the difference in the brood weight measured right after family size manipulation (when the nestlings were 6 days old) with the brood weight when the nestlings were 14 days old, (c) the brood weight when the nestlings were 14 days old, and (d) the number of fledglings produced (for further details on how we measured the four components of parental feeding effort see: Supporting Information Appendix S1).

### 2.3 The probability that females produce a late brood

Because family size manipulation is known to affect the probability of having a late brood, we quantified late broods in relation to the family size manipulation (e.g., Fokkema et al., 2017; Nicolaus et al., 2012 same population). We define a late brood here as either a repeat brood (first brood did not produce any fledglings) or a second brood (first brood did produce at least one fledgling). Females of those broods were identified during incubation while sitting tight on eggs, based on the previously applied color ring combination or later when we caught the female at the nest when the nestlings were 7 days old. Due to the fact that males were less easy to catch at late broods and only three females switched males at late broods (one from each respective manipulation group), we decided to focus our analyses on identified females at late broods only.

### 2.4 Local survival of the manipulated parents until the nest box removal experiment

**Figure 2:**
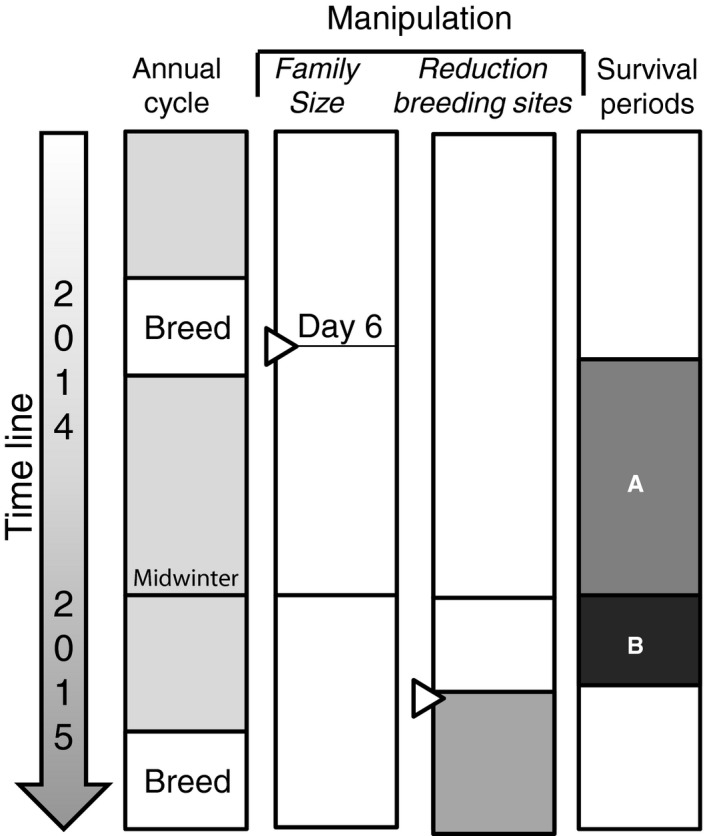
Time line of the experiment within the annual cycle. In the breeding season of 2014 when the nestlings were 6 days old, family size was experimentally manipulated (second column, black lines with triangles pointing right). Before the onset of the following breeding season in March 2015, we reduced the number of nest boxes available by 80% (third column, triangles pointing right). During the breeding season, we monitored which of the parents of the different family size manipulation groups were able to claim a breeding box. To test whether a survival cost of reproduction had already been paid before competition for nest boxes was induced, we measured the local survival probability of parents over two periods: (A) from the breeding season until midwinter and (B) from midwinter until the onset of nest box removal experiment

We needed to know which parents were alive at the moment of the nest box removal experiment to determine the effect of family size manipulation on the probability of parents of claiming a scarce nest box. Moreover, this knowledge also allowed us to estimate of the effect of family size manipulation on parental local survival until the experiment. To this end, we measured parental local survival over two different periods: (A) from the breeding season until midwinter and (B) from midwinter until the onset of our nest box removal experiment (see Figure 2). To accurately estimate the local survival probability of parents over these two periods, we used a combination of recoveries at two winter roost checks in December and March and of intensive observations of color ringed individuals in the study area during the 2 weeks preceding our nest box removal experiment (for observation protocol see: Supporting Information Appendix S1).

#### 2.4.1 Winter roost checks

Roost checks of all nest boxes in the area were performed mid‐December (period A) and mid‐March (period B; just before the nest box removal experiment see Figure. 2). During the roost check in December, all birds were taken out of the nest box and manipulated parents were identified based on their identification rings. During the roost check in March, we used handheld transponder readers (type: LID575‐ISO; manufactured by Dorset identification b.v.) to identify all roosting manipulated parents without having to open the box, handle, and potentially disturb the birds prior to the nest box removal experiment. The readers were held close to the bottom of the nest box to read the transponder identification number of the parents (previous use of these handheld transponder readers during winter roost checks in which boxes were also opened and birds taken out proved that the readers could accurately detect birds with a transponder this way).

### 2.5 The nest box removal experiment

During the 17th, 18th, and 19th of March 2015, 80% (40 of the 50) of the nest boxes present in each plot were removed. The 10 boxes left in the plot were randomly selected; they were cleaned and moved 35 m northeast from their original location to mitigate potential prior residency effects (Andreu & Barba, 2006; Harvey, Greenwood & Perrins 1979). Whenever it was not possible to shift the boxes to the northeast (e.g., no forest patches available), they were moved southeast, otherwise northwest or finally southwest. All the boxes were hung at breast height, facing east. We chose to reduce the number of boxes from 50 to 10 to induce competition in all study plots as the number of breeding Great tits per plot differed markedly (between 7 and 29 Great tit breeding pairs per study plot in the preceding four study years). In total, the number of nest boxes was reduced by this method from 600 to 121 (1 nest box was forgotten to be removed and only found later when already occupied; this box was occupied by a breeding pair of nonmanipulated parents).

The randomization procedure generated no differences between parents of the three different family size manipulation groups (reduced, control, enlarged) in terms of the distance between their original breeding box during family size manipulation and the nearest new breeding box in the following year (linear model: *F*_(2, 54)_ = 0.26, *p* = 0.77; average distance to nearest new nest box: 67 m).

#### 2.5.1 Signs of increased competition after the nest box removal

To assess whether competition increased as a consequence of the large‐scale removal of nest boxes, we (a) monitored the nest box occupation rate by dominant Great tits and subdominant Blue tits in the years previous and during the experiment, (b) quantified whether the availability of alternative natural cavities in our study area was indeed as we presumed low (see end of “introduction”) and whether Great tits were displaced from the nest boxes to these natural cavities during the experiment, and (c) measured the process of competition for the boxes in detail in one of our study plots using nest boxes which were fitted with RFID readers continuously throughout the breeding season (type: EM4102 data logger with a EM Datalog Loop Antenna of 65 mm, manufactured by: IB technology, Eccel Technology Limited; see: Supporting Information Appendix S1 for further details on the natural cavity searching protocol and the measurements with the RFID readers in the nest boxes).

### 2.6 Statistical analysis

**Figure 3:**
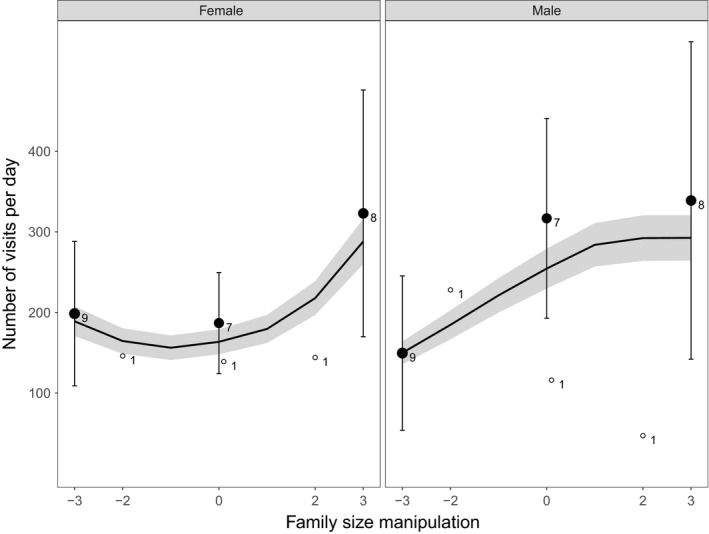
The effect of family size manipulation on the number of feeding visits per day made by the female (left part of graph) and the male (right part of graph) parents. Averages of the raw data with 95% confidence intervals (CI) are depicted with the sample size next to the points: the number of manipulated individuals for which the number of feeding visits was measured. The data points are depicted by closed black circles or white open circles to distinguish, respectively, between the majority of the family size manipulations in which three nestlings were exchanged and the minority in which two nestlings were exchanged. The black line is a predicted line calculated on the basis of the best fitting models of the whole sample. The gray‐shaded part around the predicted line is the 95% CI

**Figure 4:**
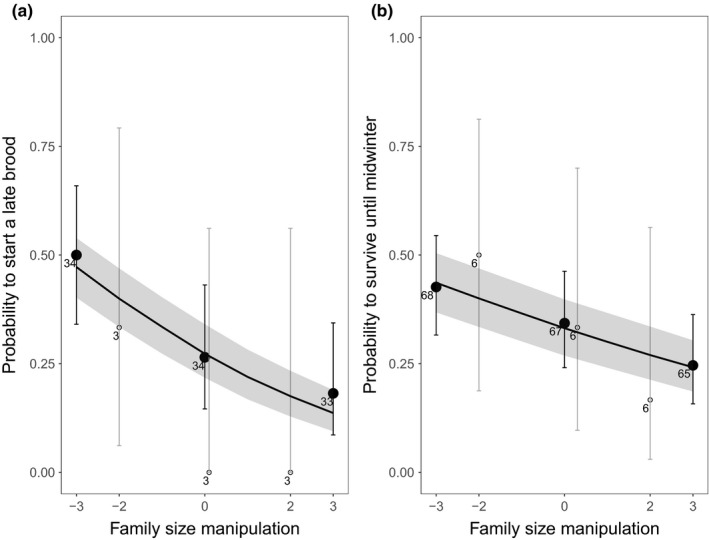
The effect of family size manipulation on (a) the probability of females to start a late brood within the same breeding season and (b) the local survival probability of manipulated male and females from breeding till midwinter. Averages of the raw data with 95% confidence intervals (CI) are depicted with the sample size next to the points: (a) the number of identified manipulated females and (b) the number of identified manipulated males and females. The data points are depicted by closed black circles or white open circles to distinguish, respectively, between the majority of the family size manipulations in which three nestlings were exchanged and the minority in which two nestlings were exchanged. The black line is a predicted line calculated on the basis of the best fitting models of the whole sample. The gray‐shaded part around the predicted line is the 95% CI

All data analysis was performed in the program R (version 3.3.1; R Core Team 2016). The mixed model analyses were performed using package lme4 (Bates, Mächler, Bolker, & Walker, 2015). Figures were created using the package ggplot2 (Wickham 2009), and the predicted lines in the Figures 2, 3, 4 with the shaded confidence interval areas were created based on predicted datasets generated using the final selected model and the predict function.

#### 2.6.1 Predictor and random variables

We included family size manipulation as a continuous variable in all analyses as we aimed to test the direction of the effect of family size manipulation (directional statistics, as in Fokkema et al., 2016). This additionally allowed us to include a quadratic effect of family size manipulation, to model any nonlinear effects (termed manipulation^2^ hereafter).

We included sex of the parent to explain variation in the number of visits made by each parent per day, in the local survival of parents until‐ and after midwinter and in the probability of parents to claim a breeding box. We included sex of the parent as main effect and in interaction with family size manipulation and manipulation^2^. To correct for nonindependence between the nests within the manipulation trios, we included a unique number as a random effect in all analyses coding for the trio of nests between which nestlings were exchanged during the family size manipulation.

#### 2.6.2 Response variables

##### Parental feeding effort

Effects of family size manipulation on the number of visits made by each parent to the nest box (54 parents at 27 broods; 9 broods not included due to incomplete measurements) and the number of fledglings produced were analyzed using a generalized linear mixed model with a Poisson error structure. We did our analysis of the number of fledglings produced including (*N* = 111 broods) and excluding all nests that failed to produce any fledglings (*N* = 93, 18 broods failed, of which 5 failed after day 14). The effect of family size manipulation on the weight change of the brood after manipulation and the brood weight at day 14 were analyzed using a linear mixed model with a Gaussian error structure. The change in brood weight could only be calculated for those broods where nestlings were still alive at day 14 (*N* = 98 of 111 broods).

##### Parental fitness components and the likelihood to claim a breeding box

Generalized linear mixed models with a binomial error structure were used to analyze variation in the probability to start a late brood, to survive until midwinter, to survive from midwinter until March and the probability of parents to claim a breeding box. The analysis of the probability that females (identified in the first broods, *N* = 110) started a late brood was based on either both repeat and second broods (*N* = 34 late broods) or second broods only (*N* = 31 late broods).

Parental local survival was calculated over two periods (see Figure 2). For period A from the breeding season (*N* = 218 identified manipulated parents) until midwinter, we considered as midwinter survivors all parents that previously raised a reduced, control, or enlarged brood that were seen during the roost check in December (*N* = 74 survivors). For period B from December to March, we considered as spring survivors all parents (of the group of parents observed roosting in December) seen during the March observations or the March roost check (*N* = 43 survivors).

We analyzed the effect of previous family size manipulation on the probability of parents to claim a breeding box in 2015 using a dataset including all 57 identified parents in the March observations and roost check.

Important to note is that 14 of the 57 parents seen in March were exclusively observed in March and not in December. These 14 parents were now not considered as alive in December in our survival analysis. We did this to keep our method of estimating the local survival of the parents comparable to the methods employed in earlier studies conducted in our study area (when we did not have the data from an additional March roost‐check and observations). Post hoc, we re‐did our survival analyses to check whether inclusion of these 14 parents exclusively seen in March as survivors in the December roost check affected the outcome of our analyses of parental survival over the two periods (Figure 2). This was not the case.

#### 2.6.3 Model selection

We used a backwards elimination procedure for model selection based on likelihood ratio tests. If included in the model, we first tested whether the interaction between manipulation^2^ and sex of the parent could be eliminated. Next we tested whether the interaction between family size manipulation and sex of the parent could be eliminated. Then, we proceeded by testing whether manipulation^2^ could be eliminated and finally we tested whether sex of the parent and family size manipulation could be eliminated. The quadratic term of family size manipulation (manipulation^2^) was never left in the model without the linear term of family size manipulation. We kept the random effect of trio‐number in the model at all times to correct for nonindependence in the dataset.

## 3 RESULTS

### 3.1 Manipulation of parental feeding effort

Family size manipulation increased parental feeding effort based on three of the four measured indexes. The number of visits per day increased in a nonlinear way with experimental family size for both males and females, but in females we found an accelerating slope, whereas in males the slope was decelerating with experimental family size (Table 2; Figure 3). We further found that the number of fledglings produced increased significantly with family size manipulation (intercept: 1.73 ± 0.06, *z* = 29.29, family size manipulation: *β* = 0.05 ± 0.02, *z* = 2.98, $\chi_{df\;1}^{2}$ = 8.86, *p* < 0.01). We found no evidence that this effect was nonlinear (manipulation^2^: *β* = 0.007 ± 0.01, *z* = 0.78, $\chi_{df\;1}^{2}$ = 0.61, *p* = 0.44). The outcome of our analysis of the effect of family size manipulation on the number of fledglings produced did not depend on whether broods that produced no fledglings were included in the sample.

**Table 2:** Outcome of the mixed model estimating the effect of family size manipulation on the number of feeding visits made by each parent (male and female) to the nest. The variance explained by the random effect trio id was 0.30

Variable	Estimate (β ± *SE*)	*z*	χ^2^	*df*	*p*
Intercept	4.96 (0.16)	30.84			
Family size manipulation	0.07 (0.005)	13.71			
Sex					
Male (relative to female)	0.46 (0.03)	14.06			
Family size manipulation^2^	0.04 (0.004)	11.81			
Family size manipulation × sex			36.51	1	<0.001
Family size manipulation × sex: male	0.05 (0.007)	6.03			
Family size manipulation^2^ × sex			208.8	1	<0.001
Family size manipulation^2^ × sex: male	−0.06 (0.004)	−14.33			

The total weight of the brood at day 14 (one week before fledging) increased significantly with family size manipulation (intercept: 118.83 ± 4.82, *t* = 24.65, family size manipulation: *β* = 7.80 ± 1.07, *t* = 7.30, $\chi_{df\;1}^{2}$ = 39.01, *p* < 0.001). Parents of the enlarged broods were thus able to maintain the higher brood weights created after manipulation. Yet, we found no effect of family size manipulation on the growth in brood weight after manipulation (intercept: 48.04 ± 3.90, *t* = 12.31, family size manipulation: *β* = −0.31 ± 1.06, *t* = −0.29, $\chi_{df\;1}^{2}$ = 0.08, *p* = 0.77). The latter results indicate that individual offspring in the enlarged broods grew less well in weight after the family size manipulation.

### 3.2 The probability to produce a late brood

We found a clear negative effect of family size manipulation on the probability of females to have a late brood during the same breeding season (Figure 4a; intercept: −0.99 ± 0.27. *z* = −3.71, family size manipulation: *β* = −0.29 ± 0.10, *z* = −2.84, $\chi_{df\;1}^{2}$ = 9.39, *p* < 0.01). We found no evidence that the effect of family size manipulation was nonlinear (manipulation^2^: *β* = 0.05 ± 0.05, *z* = 0.85, $\chi_{df\;1}^{2}$ = 0.73, *p* = 0.39). The outcome of the analysis was similar when only second broods were included in the sample.

### 3.3 The probability of parents to survive up to the next breeding season

Family size manipulation negatively affected the local survival probability of parents from the breeding season until midwinter (period A: Figure 2; results see: Figure 4b; intercept: −0.70 ± 0.16, *z* = −4.47, family size manipulation: *β* = −0.15 ± 0.06, *z* = −2.38, $\chi_{df\;1}^{2}$ = 5.85, *p* < 0.05). There was no evidence that the effect of family size manipulation was nonlinear (manipulation^2^: *β* = −0.007 ± 0.03, *z* = −0.20, $\chi_{df\;1}^{2}$ = 0.04, *p* = 0.85) nor that it differed between the sexes (family size manipulation × sex (male relative to female): *β* = −0.01 ± 0.12, *z* = −0.06, $\chi_{df\;1}^{2}$ = 0.004, *p* = 0.95; manipulation^2^ × sex: *β* = −0.02 ± 0.07, *z* = 0.24, $\chi_{df\;1}^{2}$ = 0.06, *p* = 0.81).

**Figure 5:**
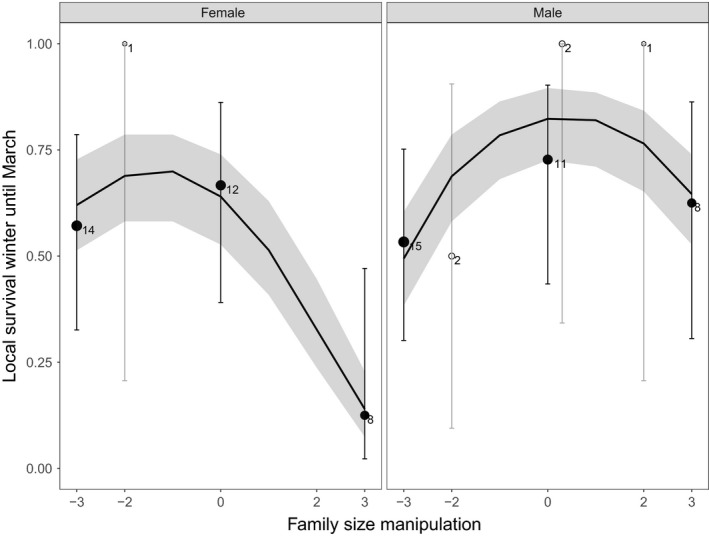
The effect of family size manipulation on the local survival probability of both parents from midwinter until the onset of our nest box removal experiment. Averages of the raw data with 95% confidence intervals (CI) are depicted with the sample size: the number of manipulated parents seen during the roost check in mid‐December. The data points are depicted by closed black circles or white open circles to distinguish, respectively, between the majority of the family size manipulations in which three nestlings were exchanged and the minority in which two nestlings were exchanged. The black line is a predicted line calculated on the basis of the best fitting models of the whole sample. The gray‐shaded part around the predicted line is the 95% CI

We further found evidence for a nonlinear negative effect of family size manipulation on the local survival probability of parents from midwinter until March (period B: Figure 2; results see: Table 3, Figure 5). The pattern in the survival effect caused by family size manipulation after midwinter was less clear. For both sexes, the effect of family size manipulation was nonlinear (manipulation^2^ × sex: *β* = 0.09 ± 0.14, *z* = 0.67, $\chi_{df\;1}^{2}$ = 0.45, *p* = 0.50), but the slope of the effect differed between the sexes, with a more pronounced negative effect in females than males (see Table 3, Figure 5).

**Table 3:** Outcome of the mixed model estimating the effect of family size manipulation on the probability of each parent to survive from midwinter until March. The variance explained by the random effect trio id was 0.43

Variable	Estimate (β ± *SE*)	*z*	*χ* ^2^	*df*	*p*
Intercept	0.60 (0.57)	1.07			
Family size manipulation	−0.39 (0.20)	−1.93			
Sex					
Male (relative to female)	0.97 (0.61)	1.58			
Family size manipulation^2^	−0.14 (0.07)	−2.00	4.47	1	<0.05
Family size manipulation × sex			4.28	1	<0.05
Family size manipulation × sex: male	0.49 (0.27)	1.86			
Rejected terms:	Family size manipulation^2^ × sex (*df* = 1)				

### 3.4 The nest box removal experiment

#### 3.4.1 Did competition increase after the nest box removal?

**Figure 6:**
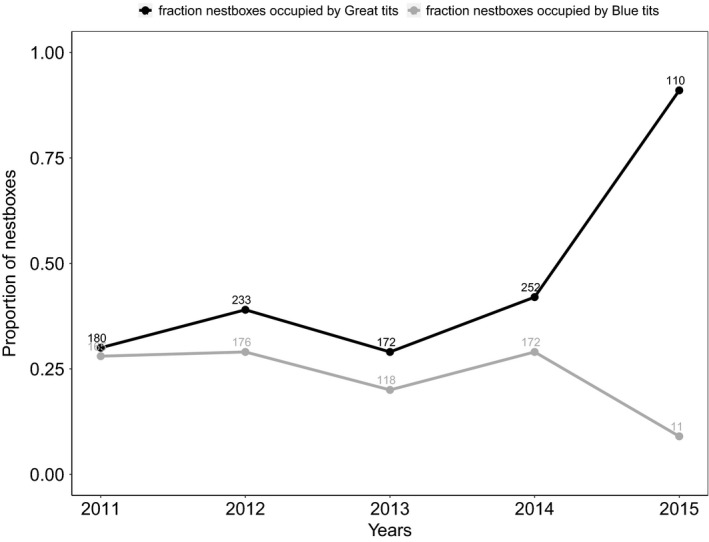
The proportion of nestboxes occupied by blue (grey line) and Great tits (black line) in the last five study years. The number of breeding pairs is depicted above the data points. From 2011 to 2014, 600 boxes were available in the area, and in 2015, the number of boxes was decreased to 121. Next to Great and Blue tits, a negligible number of breeding pairs of other species occasionally bred in the nestboxes

After the total number of boxes decreased from 600 to 121, we found that the number of Great tit breeding pairs had decreased from 252 in the previous year to 110. The remaining 11 boxes were occupied by Blue tits (Figure 6). In line with competition occurring over the boxes we found that (a) the fraction of the breeding boxes occupied by dominant Great tits increased dramatically ($\chi_{df\;1}^{2}$ = 166.25, *p* < 0.001) while the fraction occupied breeding boxes by subdominant Blue tits decreased markedly in 2015 as compared to 2011–2014 ($\chi_{df\;1}^{2}$ = 17.98, *p* < 0.001).

Furthermore (b) we found relatively few natural cavities (see: Discussion for references to other study areas) available as alternative to the nest boxes in our study area and that some competitive displacement of Great tits which formerly had bred in breeding boxes in 2014 to natural cavities in 2015 had occurred. By a combination of systematic and opportunistic checks of our study area, we detected 69 natural breeding cavities of which 50 were occupied. Seventeen cavities were occupied by Great tits (the surface area of the study area was approximately 24 km^2^; for searching protocol see: Supporting Information Appendix S1). Sixteen of the Great tits breeding in natural cavities could be identified based on existing identification rings, six of them bred in our nest boxes in 2014.

And finally (c) we found evidence based on our detailed measurements with transponder readers within one study plot that more birds visited and were interested the nest boxes than actually bred in the plot (see: Supporting Information Appendix S1). We detected 12 unique individuals with a transponder in the study plot, and all had bred in the previous year in the focal study plot, except one individual which bred in a study plot approximately 2 km away. Five of these individuals managed to claim a box for breeding in the monitored competition plot, the other seven recorded individuals with a transponder were not observed as breeder in any of the nest boxes, but did visit several (up to 6) of the available nest boxes in the plot (mostly before the first egg was laid; see: Supporting Information Appendix S1, Table S1).

#### 3.4.2 Did family size manipulation affect the probability to claim a scarce nest box?

**Figure 7:**
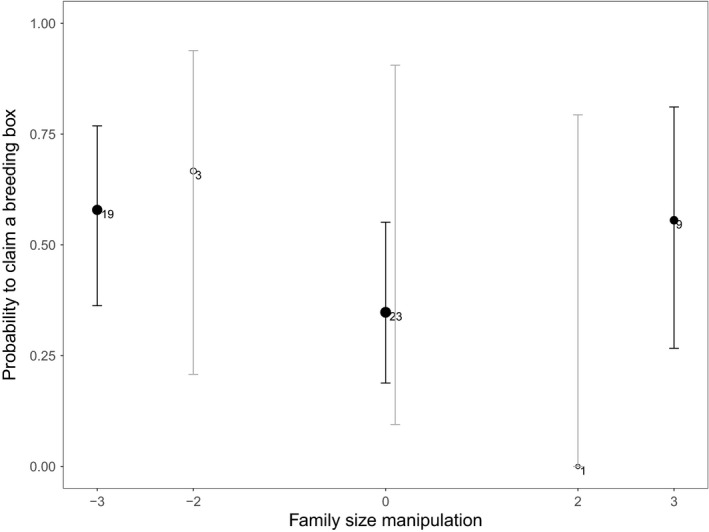
The effect of experimental family on the probability of parents to claim a scarce breeding box the following spring after the large‐scale removal of nest boxes. Family size manipulation did not affect the probability of parents to claim a breeding box next spring. Averages of the raw data with 95% confidence intervals (CI) are depicted with the sample size next to the points (sample sizes correspond to the number of parents seen just before the onset of the experiment in March). The data points are depicted by closed black circles or white open circles to distinguish, respectively, between the majority of the family size manipulations in which three nestlings were exchanged and the minority in which two nestlings were exchanged

In contrast to our expectation, family size manipulation had no effect on the probability of parents to claim a breeding box the following spring after competition was induced (Figure 7; intercept: −0.43 ± 0.28, *z* = −1.50, family size manipulation: *β* = −0.06 ± 0.13, *z* = −0.42, $\chi_{df\;1}^{2}$ = 0.18, *p* = 0.67). We also did not find any evidence for nonlinear (manipulation^2^: *β* = 0.14 ± 0.08, *z* = 1.63, $\chi_{df\;1}^{2}$ = 3.27, *p* = 0.07) or sex‐specific effects of family size manipulation (family size manipulation × sex (male relative to female): *β* = 0.33 ± 0.39, *z* = 0.84, $\chi_{df\;1}^{2}$ = 0.75, *p* = 0.39; manipulation^2^ × sex: *β* = 0.01 ± 0.15, *z* = 0.07, $\chi_{df\;1}^{2}$ = 0.006, *p* = 0.94)), nor for differences between males relative to females (*β* = 0.80 ± 0.68, *z* = 1.32, $\chi_{df\;1}^{2}$ = 2.04, *p* = 0.15).

## 4 DISCUSSION

We set out to test whether family size negatively affects the competitive ability of parents in later life. We experimentally manipulated the family size that Great tit parents had to raise and in the next breeding season induced competition among the parents for breeding sites via a large‐scale nest box removal experiment. If indeed reproductive effort negatively affects future parental competitive ability this could explain how the occurrence of cost of reproduction depends on both individual reproductive investment and the competitive environment (Nicolaus et al., 2012). It would imply that individual fitness is not only the product of individual reproductive decisions, but also of the reproductive decisions (and hence the competitive ability) of other individuals within the same population (Both, Visser, & Verboven, 1999; Mesterton‐gibbons & Hardy, 2004; Packer & Pusey, 1995; Svensson & Sheldon, 1998; Wilson, 2014).

Our expectation was that the survival of parents who raised experimentally manipulated families would not be affected until the onset of our nest box removal experiment, based on the finding of Nicolaus et al. (2012) that a survival cost of reproduction in our population was only paid under competition after midwinter. We expected that differences in parental competitive ability due to our family size manipulation would therefore show up in the likelihood of parents to claim a scarce nest box after competition was induced. In contrast to our expectation, we found no evidence that experimental family size negatively affected the ability of Great tit parents to compete for nest boxes the following spring. However, also in contrast to our expectation, we did find a clear overwinter survival effect of family size manipulation before the onset of our experiment. Below we discuss potential reasons for the observed effects and emphasize that competition may affect why in some cases costs of reproduction are found, and not in others.

### 4.1 Did family size manipulation affect parental feeding effort?

One reason, why we observed no effects of experimental family size on parental competitive ability could be that we were unsuccessful in manipulating parental feeding effort and/or induce competition among the parents in the following spring. We conclude, however, that parental feeding effort did increase with experimental family size. Parents raising larger experimental broods increased their number of feeding visits and were able to sustain the higher number of nestlings and brood weight until fledging. The fact that the increase in the number of feeding visits at least for the males leveled off at the enlarged broods and that the growth in brood weight after manipulation was not affected by experimental family size indicates that parents did not completely keep up with the increased demand of their brood (Tinbergen and Verhulst 2000).

### 4.2 Was competition for breeding boxes successfully induced?

Further, we conclude that competition also did increase as a consequence of the nest box removal experiment. First of all unlike previous years, after the nest box removal experiment, all nest boxes were occupied (Figure 6). The vast majority of the boxes were occupied by Great tits, but less than half of the breeding pairs around in the last year managed to claim a spot. Overall, the fraction of nest boxes occupied by dominant Great tits in the breeding season drastically increased and the fraction of nest boxes occupied by subdominant Blue tits decreased relative to the expected numbers from previous years. This is in line with the experimental findings of Dhondt and Adriaensen (1999) and Löhrl (1977), who showed that Great tits outcompeted Blue tits from breeding boxes.

Secondly, our transponder data gathered in one of the study plots, showed that more Great tits were interested in the boxes, than that could actually breed in them, indicating that there was competition for the nest boxes (Supporting Information Appendix S1, Table S1). Taking into account that only the manipulated parents of the year before had a transponder and first year breeders which did not have a transponder were also around, the true degree of competition was likely higher. It is known from other passerine species that birds also under “natural” conditions exhibit prospecting behaviour, that is, inspecting multiple nesting sites before and during breeding (Doligez, Cadet, Danchin, & Boulinier, 2003; Doligez, Pärt, & Danchin, 2004; Pärt & Doligez, 2003; Sánchez‐Tójar et al., 2017). For House sparrows (*Passer Domesticus*), it was shown that experienced adult breeders prospected very little, likely because they hold on to the same territories year round (Sánchez‐Tójar et al., 2017). The same may hold for the territorial Great tit (Andreu & Barba, 2006; Tinbergen, 2005) under “natural” levels of competition for nest boxes. We have no data of previous study years on visits of transpondered birds to the nest boxes in the breeding season to judge this. However, the pattern in our study, as measured from the transpondered parents that bred in the area the year before, is consistent with competition resulting in the increased number of visits. In particular, individual Great tits that visited multiple boxes did not manage to claim a breeding box later, whereas those that did claim a box later concentrated on this box all the time. This suggests that the parents that showed interest in multiple nest boxes did so because they had no breeding place yet of their own (see: Supporting Information Appendix S1, Table S1).

Thirdly, our inventory of the availability and use of natural cavities showed that relatively few natural cavities were available and that competitive displacement of Great tits to natural cavities had occurred. In total, we found 69 suitable natural cavities of which 50 were occupied over the whole study area. Relative to other study systems this is a low natural cavity availability (see: e.g., Cockle, Martin, & Drever, 2010; Lõhmus & Remm, 2005; Maziarz, Wesołowski, Hebda, & Cholewa, 2015; Newton, 1994; Robles, Ciudad, & Matthysen, 2011). It indicates, however, that next to the 121 nest boxes remaining after competition was induced, some alternative breeding places were available to the tits. We found that at least six Great tit parents that bred in nest boxes in 2014 (of the 252 breeding pairs, 600 nest boxes) switched to using natural cavities in the 2015 breeding season after competition for nest boxes was drastically induced. Nest boxes are generally preferred by Great tits over natural cavities (Drent, 1987; Lõhmus & Remm, 2005), we would thus expect that the Great tits that switched from using a nest box to using a natural cavity did so because they were locally outcompeted.

### 4.3 Were fitness costs of reproduction already paid before the experiment?

There may be both temporal and spatial variation if, when and how costs of reproduction are being paid. Based on our fitness measures in this study, costs of reproduction were paid in two ways: (a) by parents with experimentally enlarged families foregoing to start a late brood within the same season (Figure 4a) and (b) via survival cost of reproduction (Figures 4b and 5). We judge that the effects of family size manipulation on our measures of parental local survival were the result of actual mortality rather than emigration because local survival effects took place before our nest box reduction experiment. Great tits are known once they have settled and bred for the first time to be very site faithful (Andreu & Barba, 2006; Tinbergen, 2005). Furthermore, from the observations we did in the area prior and after the experiment (see: Supporting Information Appendix S1), we have no indication that parents depending on their family size manipulation selectively left the area after the onset of our experiment (proportion of parents seen per manipulation group of the total number of parents observed, respectively, before or after the onset of the experiment; before: *N* = 47, R: 0.34, C: 0.47, E: 0.19; after: *N* = 40, R: 0.35, C: 0.43, E: 0.23).

We suggest that in this particular year, due to the fact that the survival cost of reproduction was already paid before we induced competition for nest boxes, any competitive differences among the parents resulting from the previous family size manipulation may have already been erased before the next spring. The observed negative effect of family size manipulation on the probability of parents to start a late brood within the same season could have exerted an additional compensatory effect. From previous work, we know, however, that even though similar negative effects of family size manipulation on the probability of parents to start a late brood occurred, long‐term effects on parental survival (this study, Fokkema et al., 2017; Nicolaus et al., 2012) and parental competitive ability in spring still persisted (Fokkema et al., 2016).

The observation that a survival cost of reproduction was mostly paid in our experimental year before midwinter under nonmanipulated control levels of competition (Figure 4b) is in contrast to previous family size manipulation studies in our study area (Fokkema et al., 2016, 2017; Nicolaus et al., 2012). A possible reason related to competition is that the availability of an important winter food for Great tits in our study area, Sea buckthorn (*Hippophae rhamnoides*) berries, was exceptionally low in the winter of 2014. This potentially resulted in increased competition. Local annual survival of Great tits in our study area correlates positively with the winter density of Sea buckthorn berries (see: Supporting Information Appendix S1). In line with the scenario of a high level of competition, the breeding population of Great tits in 2014 was exceptionally high (252 breeding pairs; previous 4 years: mean ± SD: 206 ± 35 pairs) and the abundance of Sea buckthorn berries (2014/2015) exceptionally low. Tinbergen et al. (1985) suggested that only in years with a low winter food Great tit parents paid a survival cost of reproduction. Perhaps, due to the very low availability of Sea buckthorn berries in our study year, selective disappearance of individuals with low competitive ability already occurred during the winter, and as a consequence we found no effect of previous reproductive effort on the competition for the scarce nest boxes the following spring.

## 5 CONCLUSIONS

In contrast to previous work (Fokkema et al., 2016; Siefferman & Hill, 2005a), we found no evidence for a negative effect of family size on future parental competitive ability in the competition for scarce breeding sites. We did find that costs of reproduction were paid in our experiment both in terms of fecundity and survival costs before the onset of the competition experiment. Our data indicate that we successfully manipulated (a) parental feeding effort and (b) the level of competition among the parents the following spring. Perhaps, in this particular year, differences in parental competitive ability due to previous reproductive effort did play a role earlier in the winter, due to high competition over scarce winter food. The less competitive birds may as a consequence have already been eliminated from the population before our experiment took place. Whether and when costs of reproduction are paid may thus depend on the level of competition parents encounter later in their life. There is a great need for studies manipulating both reproductive effort and the subsequent level of competition, to establish these effects.

## CONFLICT OF INTEREST

None declared.

## AUTHOR CONTRIBUTIONS

RF, RU, JM, and CB conceived the ideas and designed the methodology. RF, RU, JM, CB, and LF collected the data; RF analyzed the data; RF and JM led the writing of the manuscript. All authors contributed critically to the drafts and gave final approval for publication.

## DATA ACCESSIBILITY

Data available from the Dryad Digital Repository: https://doi.org/10.5061/dryad.3cn644h.
