# Supplementary material for: Reproductive effort and future parental competitive ability: A nest box removal experiment
Source: Ecol Evol. 2018 Aug 11;8(17):8865–79. doi: 10.1002/ece3.4342 (PMC6157679; doi:10.1002/ece3.4342)
Supplement: Supplementary file 1 [file ECE3-8-8865-s001.docx]

## Appendix S1

### Measuring components of parental feeding effort

We measured the effect of family size manipulation on parental feeding effort based on four components: 1) the number of visits made per day by each parent to the nest box, 2) the difference in the brood weight measured right after family size manipulation (when the nestlings were 6 days old) with the brood weight when the nestlings were 14 days old, 3) the total produced brood weight and 4) the number of fledglings produced.

To measure the visits made by each parent to the nest box, we made use of an RFID transponder ring (type: EM4102 bird PIT tag 2.6mm, manufactured by: IB technology, Eccel Technology Limited). Some parents that raised a manipulated brood had already been provided with a transponder ring in 2013 (N=26) for a different study, but most had none. Therefore we caught all parents (including the parents previously provided with a transponder) in the nest box using spring traps the day after family size manipulation. In case we failed to catch one or both of the parents we tried again two days later. In total we caught and provided 208 of the 222 parents that raised manipulated broods with a transponder ring (10 parents were either caught but forgotten to be provided with a transponder ring or failed to be caught but identified with binoculars on the basis of existing identification rings; four parents could not be caught or identified based on existing identification rings). Transponder rings were applied on the left or right leg of the parent, on the other leg an aluminium ring with unique inscription and a coloured plastic ring were attached in a unique combination. To measure the number of visits made by each parent to the nest box we used transponder readers (type: LID665, version V804, manufactured by Dorset identification b.v.). A dummy antenna mimicking the real antenna of the transponder readers was fitted on the inside of the box around the entrance hole when the nestlings were 10 days old to get the birds acquainted to what was in effect a novel object. Next at day 11, we replaced the dummy antenna with the real antenna fitted to the reader and measured the number of feeding visits made by each parents during the whole following day (day 12; age around which brood energy demand peaks: van Balen 1973; Tinbergen and Dietz 1994; Sanz and Tinbergen 1999; measurements during 24 hours). From this data, we calculated the number of feeding visits made by each parent during the whole day (following: Nicolaus et al. 2012 and Fokkema et al. 2016).

To measure the change in weight of the brood after family size manipulation we weighed the brood two times, one time right after family size manipulation (nestlings 6 days old) and the other time when the nestlings were 14 days old. The brood weight measured when the nestlings were 14 days old was additionally used in our analysis of the effect of family size manipulation on the total brood weight produced. The number of fledglings produced was determined by daily fledge checks of the manipulated broods 21 days after hatching. When the brood had fledged, nest material was carefully inspected for any dead nestlings. The number of fledglings was determined by subtracting the number of nestlings seen when the nestlings were 14 days old with any dead nestlings found later in the nest.

### Observation protocol to determine the surviving parents in March

Starting two weeks preceding the nest box removal experiment, we conducted observations to identify all parents who had previously raised a manipulated brood, based on their existing unique colour ring combination. We worked with a team of 4 observers. We visited each of the 12 selected study plots, 9 of the areas were visited 4-6 times and 3 of the areas 2-3 times. The latter areas were visited less because we expected less manipulated parents based on the distribution of family size manipulations in 2014 and the December roost check at midwinter. Each observation session within a plot lasted 4-6 hours, during which we walked through the whole area systematically. Birds were located based on their calls and songs. To provoke the birds to call or sing we played back Great tit calling sounds and songs. Once birds were spotted, we attempted to identify them using binoculars (8x, 10x), a telescope (20-60x or 15-45x) and/or a camera with a long-focus lens (400mm) on the basis of their colour ring combination (see section above). The exact location of the birds was noted down in GPS-coordinates using a GPS device (Garmin GPSmap 60 or 62s) or map. Observations were also continued with a team of 2 observers after the experiment until the beginning of May, to monitor what was happening after we induced competition for nest boxes. The latter gathered observation data was not used for estimating the survival of the parents.

### Measuring whether competition was induced:

#### Locating and identifying the inhabitants of natural cavities

We checked the study area for breeding attempts in natural cavities during the whole breeding season (see accompanying article for more information on the study area). We conducted systematic checks of the 8 study plots in which most manipulated parents were seen preceding the experiment (see section above). We systematically surveyed the wood lots with either 1 or 2 persons. We located natural cavities in tree trunks or in branches by visual inspection of trees, hereby also making use of sounds made by birds inhabiting these cavities (alarm calls, singing or begging calls of the offspring). We played back these sounds while walking through the woodlots to provoke birds to respond (see section above). For 3 of these 8 study plots we additionally systematically surveyed (using a similar method as described above) woodlots adjacent to the study plots (within a distance of 1 kilometre from the plot) where we suspected that the trees in these areas would have natural cavities (trees were older and bigger and more dead trees/branches were present, e.g. Newton 1994). Besides these systematic checks, in an opportunistic way during our normal rounds of the whole area we also paid extra attention to the presence of natural cavities in the remaining 4 study plots and the woody areas between our study plots.

If natural cavities were located we used a ladder and an endoscope (type: Basetech BSK-100) to determine if the cavity was occupied and, if possible, by which species or, in the case of Great tits, by which individual birds (based on existing identification rings). If unattended Great tit broods (based on the appearance of the eggs) were detected we attempted to identify the breeding birds from a distance with binoculars based on their existing identification rings.

In total, we detected 69 breeding cavities of which 50 were occupied. 18 were occupied by Great spotted woodpeckers (*Dendrocopos major*), 17 by Great tits, 7 by Blue tits, 1 by a Spotted flycatcher (*Muscicapa striata*), 1 by the Common redstart (*Phoenicurus phoenicurus*), while of 6 cavities the species of the breeding pair could not be identified (the surface area of the study area was approximately 24 km^2^). 16 Great tits breeding in the detected natural cavities could be identified based on existing identification rings, 6 of them bred in our nest boxes in 2014, 5 individuals were first year breeders fledged from one of the nest boxes in 2014, 3 fledged from our boxes before 2014 and 2 had been detected in our nest boxes during winter roost checks in 2013 but were never detected in the breeding season. The 19 non-occupied cavities were judged as potentially suitable breeding cavities and had an estimated depth of minimally 7 cm measured from below the entrance until the bottom of the cavity (average depth: 15 cm; cavity depth of occupied natural cavities in the Białowieza forest in Poland ranged from 3 to 109 cm, see: Maziarz, Wesołowski, Hebda, & Cholewa, 2015).

#### Monitoring competition over the new nest boxes

We measured the process of competition for the boxes in detail in one of our study plots using boxes which were fitted with RFID readers continuously throughout the breeding season (type: EM4102 data logger with a EM Datalog Loop Antenna of 65mm, manufactured by: IB technology, Eccel Technology Limited). We detected 12 unique individuals with a transponder in the study plot, all had bred in the previous year in the focal study plot, except one individual which bred in a study plot approximately 2 km away (see: Table S1). Five of these individuals managed to claim a box for breeding in the monitored competition plot, the other seven recorded individuals with a transponder were not observed as breeder in any of the nest boxes, but did visit several (up to 6) of the available nest boxes in the plot (mostly before the first egg was laid).

### Annual local survival and Sea buckthorn berries

During the month of December in the winters of 2008-2013 the number of berries was estimated on 19 representative Sea buckthorn shrubs (*Hippophae rhamnoides*) in the center of the study area. These berries form a prime winter food for the Great tits in our study area (Vollmer et al. 2007). Using a generalized linear mixed model with study year as a random factor we tested the relationship between the annual local survival of parents and the average availability of Sea buckthorn berries per shrub in December. In addition, we included the effect of sex of the parent and the interaction between sex of the parent and the average availability of berries on the local survival of parents. We found that the availability of Sea buckthorn berries in December had a marked positive effect on Great tit annual local survival, with a more pronounced positive effect in males than for females (Table S2). In our focal study year, 2014, the availability of Sea buckthorn berries was for logistical reasons counted only in November. At this point the average availability of berries per shrub was already lower than the availability in December in all the other study years (2014 mean ± SD: 544 ± 950 berries; 2008-2013: 6492 ± 3519). The lowest number of berries recorded in the previous study years before 2014, was in December 2012 (490 ± 827 berries).

### References

van Balen JH. 1973. A Comparative Study of the Breeding Ecology of the Great Tit *Parus Major* in Different Habitats. Ardea 61:1–93.

Fokkema RW, Ubels R, Tinbergen JM. 2016. Great tits trade off future competitive advantage for current reproduction. Behavioral Ecology 27:1656–1664.

Maziarz M, Wesołowski T, Hebda G, Cholewa M. 2015. Natural nest-sites of Great Tits (*Parus major*) in a primeval temperate forest (Białowieza National Park, Poland). Journal of Ornithology 156:613–623.

Newton I. 1994. The role of nest sites in limiting the numbers of hole-nesting birds: A review. Biological Conservation 70:265–276.

Nicolaus M, Michler SPM, Ubels R, van der Velde M, Bouwman KM, Both C, Tinbergen JM. 2012. Local sex ratio affects the cost of reproduction. Journal of Animal Ecology 81:564–572.

Sanz JJ, Tinbergen JM. 1999. Energy expenditure, nestling age, and brood size: an experimental study of parental behavior in the great tit *Parus major*. Behavioral Ecology 10:598–606.

Tinbergen JM, Dietz M. 1994. Parental energy expenditure during brood rearing in the Great Tit (*Parus major*) in relation to body mass, temperature, food availability and clutch size. Functional Ecology 8:563–572.

Vollmer A, Both C, Tinbergen J. 2007. Duindoornbessen als wintervoedsel van de Koolmees. Limosa 80:68–75.

### **Tables and table legend**s

**Table S1:** Overview of the number of visits made by individual Great tits provided with transponder rings to nest boxes, in one of our competition plots, throughout the breeding season. The first two columns depict the individual and its sex respectively. The next 10 columns depict each nest box in the study plot and the values in the cells represent the number of visits each individual made to that particular box. All nest boxes depicted here were occupied by breeding pairs, but not all individuals had a transponder. If the cells are highlighted this means that this was the box in which the individual was observed breeding. Note that individuals that did not breed in any of the boxes in the study area visited different boxes. For those individuals we have depicted when the visits took place in the last three columns: before the onset of laying, after the onset of laying and the date of the last registration of the bird in one of the boxes in the plot (the average laying date of first broods in the monitored plot was 28/04/15). *Individual 1 bred in two nest boxes, her first brood in box 6 failed and she laid a repeat clutch in box 8. She and her partner (without a transponder) were however then displaced while breeding by the former owners that started a second brood in box 8.

|  |  | Nest box | | | | | | | | | | Timing of visits | |  |
| --- | --- | --- | --- | --- | --- | --- | --- | --- | --- | --- | --- | --- | --- | --- |
| ID | sex | 1 | 2 | 3 | 4 | 5 | 6 | 7 | 8 | 9 | 10 | visit before clutch initiation | visit after clutch initiation | date last registration |
| 1* | F |  |  |  |  |  | 390 |  | 122 |  |  |  |  |  |
| 2 | M |  |  |  |  | 986 |  |  |  |  |  |  |  |  |
| 3 | F |  |  | 1 | 4 | 1 |  |  | 1 | 2 | 2 | 11 | 0 | 15/03/15 |
| 4 | M |  |  |  |  |  |  | 168 |  |  |  |  |  |  |
| 5 | M |  |  |  |  | 1 | 15 |  |  | 6 | 12 | 16 | 18 | 10/05/15 |
| 6 | F | 1 |  |  | 63 |  | 12 | 79 |  |  |  | 148 | 7 | 06/05/15 |
| 7 | F |  |  |  |  |  |  | 1759 |  |  |  |  |  |  |
| 8 | M |  | 6 |  |  | 1 | 1 |  |  |  |  | 6 | 2 | 03/05/15 |
| 9 | M |  |  |  | 1862 |  |  |  |  |  |  |  |  |  |
| 10 | F |  | 1 |  |  | 4 |  |  |  | 170 | 26 | 195 | 6 | 22/05/15 |
| 11 | F | 7 | 2 | 1 |  |  |  | 2 | 3 | 13 |  | 8 | 20 | 09/05/15 |
| 12 | F |  | 6 | 1 | 1 |  |  |  |  |  |  | 7 | 1 | 30/04/15 |

**Table S2:** Outcome of the mixed model used to estimate the relationship between the sex specific local annual survival of Great tit parents in the period from 2008-2013 and the availability of Sea buckthorn (*Hippophae rhamnoides*) berries assessed in December .

| Variable | Estimate (β±SE) | z | Chi2 | d.f. | p |
| --- | --- | --- | --- | --- | --- |
| intercept | -0.81 (0.10) | -8.57 |  |  |  |
| Sea buckthorn berries | 0.31 (0.10) | 3.12 |  |  |  |
| sex |  |  |  |  |  |
| male (relative to female) | -0.19 (0.10) | -1.89 |  |  |  |
| Sea buckthorn berries x sex |  |  | 4.57 | 1 | < 0.05 |
| Sea buckthorn berries x sex: male | 0.24 (0.11) | 2.12 |  |  |  |
